# Screening of miRNAs in plasma as a diagnostic biomarker for cardiac disease based on optimization of extraction and qRT-PCR condition assay through amplification efficiency

**DOI:** 10.1186/s12896-021-00710-w

**Published:** 2021-08-16

**Authors:** Eunmi Ban, Haejin Kwon, Hong Seog Seo, Young Sook Yoo, Eun Joo Song

**Affiliations:** 1 College of Pharmacy and Graduate School of Pharmaceutical Sciences, Ewha Womans University, Seoul, 03760 Republic of Korea; 2 Cardiovascular Center, Korea University Guro Hospital, Korea University Medicine, Seoul, 08308 Republic of Korea; 3 Molecular Recognition Research Center, Korea Institute of Science and Technology, Seoul, 02792 Republic of Korea

**Keywords:** qRT-PCR, miRNA, Amplification efficiency, Biomarker, Plasma

## Abstract

**Background:**

Although quantitative real-time PCR (qRT-PCR) is a common and sensitive method for miRNAs analysis, it is necessary to optimize conditions and minimize qRT-PCR inhibitors to achieve reliable results. The aim of this study was to minimize interference by contaminants in qRT-PCR, maximize product yields for miRNA analyses, and optimize PCR conditions for the reliable screening of miRNAs in plasma.

**Methods:**

The annealing temperature was first optimized by assessing amplification efficiencies. The effects of extraction conditions on levels of inhibitors that interfere with PCR were evaluated. The tested extraction conditions were the volume of the upper layer taken, number of chloroform extractions, and the inclusion of ethanol washing, a process that reduces PCR interference during RNA extraction using TRIzol.

**Results:**

An acceptable amplification efficiency of RT-qPCR was achieved by the optimization of the annealing temperature of the tested miRNAs and by the collection a supernatant volume corresponding to about 50% of the volume of TRIzol with triple chloroform extraction. These optimal extraction and PCR conditions were successfully applied to plasma miRNA screening to detect biomarker candidates for the diagnosis of acute myocardial infarction.

**Conclusion:**

This is the first study to optimize extraction and qRT-PCR conditions, while improving miRNA yields and minimizing the loss of extracted miRNA by evaluations of the amplification efficiency.

## Background

MicroRNAs (miRNAs) are small, non-coding RNAs that function in RNA silencing and the regulation of gene expression [1, 2]. Consequently, miRNAs play roles in many diseases, including cancer and diabetes. In addition, miRNAs are important biomarkers for clinical diagnosis and therapeutics [3, 4]. Screening for miRNAs in cells, tissues, and biological fluids, including plasma and urine, is performed as part of many clinical and biological studies. Circulating miRNAs in the serum or plasma have great promise as non-invasive biomarkers for the early diagnosis of various diseases owing to their ease of access and long-term stability [5].

Quantitative real-time PCR (qRT-PCR) is typically used to analyze miRNAs in the serum or plasma. qRT-PCR has high sensitivity and can detect low-abundance miRNAs in these sample types. In qRT-PCR, complementary DNA (cDNA) is first synthesized from mRNA or total RNA by reverse transcription. The cDNA is then used as a template for PCR and amplified. The amplification efficiency is usually calculated using template dilution series by plotting Cq values against the log of the template amount. However, cDNA amplification by PCR is often restricted by various issues, such as an ineffective annealing temperature or the presence of PCR inhibitors [6]. These factors can dramatically reduce the sensitivity and amplification efficiency of PCR, leading to the misinterpretation of results and a low reproducibility [7].

For these reasons, PCR and RNA extraction conditions should be optimized to eliminate interfering molecules. The optimization process should be based on assessments of amplification efficiency to reflect actual PCR conditions, and a method for obtaining reliable and reproducible PCR results should be developed. Evaluations of amplification efficiency are particularly important for analyses of miRNAs in plasma because the miRNA concentrations are very low and the reaction can be affected by sample matrix and contaminants.

This was a pilot study aimed at optimizing sample extraction and qRT-PCR conditions for reliable endogenous miRNA analyses in plasma. For this study, we first assessed the amplification efficiency of PCR. The annealing temperature and extraction conditions were evaluated and optimized to minimize inhibitors of PCR. The optimal extraction and PCR conditions were applied to detect miRNAs in plasma samples from control individuals and patients with acute myocardial infarction (AMI) in order to evaluate candidate diagnostic biomarkers.

## Methods

### Chemicals and materials

All the primers were purchased from Cosmogenetech (Seoul, Korea). Synthetic miRNA-16 (5′-UAGCAGCACGUAAAUAUUGGCG-3′), miRNA-21 (5′-UAGCUUAUCAGACUGAUGUUGA-3′), miRNA-132 (5′-UAACAGUCUACAGCCAUGGUCG-3′), miRNA-155 (5′-UUAAUGCUAAUCGUGAUAGGGGUU-3′), and miRNA-499 (5′-UUAAGACUUGCAGUGAUGUUU-3′) was purchased from ST Pharm (Seoul, Korea). Synthetic C. elegans miRNA-39-1 (Cel-miR-39-1) (5′-UCACCGGGUGUAAAUCAGCUUG-3′) was purchased from Qiagen (Fredrick, MD, USA). Trizol LS reagent and DNase/RNase free water were obtained from Invitrogen (Carlsbad, CA, USA). Glycogen (Invitrogen, San Diego, CA, USA) and yeast tRNA (Sigma-Aldrich, St. Louis, MO, USA) were purchased from Invitrogen and Sigma-Aldrich, respectively.

### MiRNA extraction from plasma

After 100 µL of commercial pooled human plasma collected from six healthy donors (BioChemed Services, Winchester, VA, USA) or samples from healthy individuals or patients (Cardiovascular Center of Korea University Guro Hospital) and 750 µL of TRIzol LS reagent (Invitrogen, Carlsbad, CA, USA) mixed, synthetic Caenorhabditis elegans miRNA-39 (cel-miR-39) was spiked into sample, followed by the addition of 200 µL of chloroform. Each sample was vortexed for 30 s and incubated at room temperature for 5 min. Phase separation was performed by centrifugation at 12,000×*g* for 15 min at 4 °C. Then 400 µL of the aqueous phase was transferred to a new tube. For triple chloroform extraction, after the aqueous phase was transferred to a new tube, add an equal volume of chloroform, vortex 30 s and centrifuge at 12,000×*g* for 15 min, transfer the aqueous to a fresh tube. And this chloroform extraction step was repeated once more. Then, 500 µL of isopropanol was added to the aqueous phase and 5 mg/mL glycogen (Invitrogen) and 100 ng/µL yeast tRNA (Sigma-Aldrich, St. Louis, MO, USA) were added for RNA precipitation. After incubation at –80 °C for 1 h, samples were centrifuged at 20,000×*g* for 30 min at 4 °C. For washing, 700 µL of 70% ethanol was added to the RNA pellet. The washed sample was vortexed for 30 s and centrifuged at 7500×*g* for 5 min at 4 °C. Finally, the washed or unwashed RNA pellet was dissolved in 30 µL of RNase-free water (Invitrogen) and the total RNA concentration and residual phenol level were evaluated using a NanoDrop One Spectrophotometer (Thermo Scientific, Waltham, MA, USA) with the Acclaro Contaminant Identification (ID) feature, which can detect protein, phenol, and guanidine salts in dsDNA and RNA samples by measuring absorbance at 260 nm.

### Quantitative real-time PCR

Isolated RNAs and miRNAs were used for cDNA synthesis. Transcription was performed in a reaction mixture containing 500 ng of RNA sample using the Mir-X miRNA First Strand Synthesis and SYBR qRT-PCR Kit (Clontech, Mountain View, CA, USA) according the manufacturer’s protocol. cDNA synthesis was conducted for 1 h at 37 °C and the reaction was terminated by heating at 85 °C for 5 min. cDNA was mixed with SYBR qPCR Mix containing 0.5 μL of 10 μM miRNA-16-, 21-, 132-, 155-, and 499-specific primers and the universal mRQ 3′ reverse primer from the Mir-X miRNA qRT-PCR SYBR Kit (Clontech). PCR was performed for 10 s at 95 °C; 5 s at 95 °C, and 20 s at 60 °C for 40 cycles and finalized by a melting curve analysis with 5-s intervals for each 0.5 °C. The CFX Connect Real-time PCR Detection System (Bio-Rad, Hercules, CA, USA) was used for both cDNA synthesis and real-time PCR. Finally, the samples were analyzed using CFX Manger (Bio-Rad). All miRNA expression levels were normalized to the cel-miR-39 expression. For the optimization of qRT-PCR and extraction conditions for exogenous and endogenous miRNAs, standard curves were generated to calculate the qRT-PCR efficiency using tenfold serial dilutions of cDNA templet generated from synthetic miRNAs or extracted RNA samples from plasma. Triplicate standard curves were included in all qPCR assays. The mean Cq values were plotted against the logarithm of the dilution factor of cDNA templet, and both the R^2^-value and PCR efficiency for each assay were determined from the respective plots. The amplification efficiency was calculated using the following formula: efficiency (%) = {[10(− 1/slope)] − 1} × 100.

### Study subjects and clinical characteristics

Patients with AMI admitted to the Cardiovascular Center of Korea University Guro Hospital were consecutively recruited for 4 years. AMI was defined as a typical increase and gradual decrease of biochemical markers of myocardial necrosis, with at least one value above the 99th percentile upper reference limit for creatine kinase-MB (CK-MB) (i.e., > 0.1 ng·mL-1) and CK-MB (i.e., > 6.73 ng·mL-1) and at least one of the following: ischemic symptoms, ECG changes indicative of new ischemia (new ST-T changes or new left bundle branch block [LBBB]), development of pathologic Q waves on ECG, and imaging evidence of new loss of viable myocardium or new regional wall motion abnormality. Control subjects who did not have cardiovascular disease were recruited from individuals undergoing a routine health check-up at the Cardiovascular Center of Korea University Guro Hospital. For the control group, participants were excluded if they had a history of cardiovascular disease (myocardial infarction, unstable angina, stroke, or cardiovascular revascularization), malignancy, or severe renal or hepatic disease. In this study design, the groups were matched with respect to age and sex. In particular, individuals in the control and AMI groups were restricted to men aged 40–60 years because heart disease is age-related. In addition, individuals with diseases other than AMI, such as hypertension, diabetics, and dyslipidemia, were excluded from both groups. As a result of these strict criteria, the sample size was small and differed between the two groups. Plasma samples were ultimately collected from four male patients with AMI aged 52–60 years (55.3 ± 4.2 years) and from three control males aged 52–59 years (57.0 ± 3.4 years).

All participants provided written informed consent and the study was performed in accordance with the Declaration of Helsinki of the World Medical Association and approved by the Korea University Institutional Review Board (KUGH-12118). The blood samples were collected from the antecubital vein of the forearm. Each sample was immediately stored at − 80 °C for subsequent assays.

### Statistics

Statistical analyses were performed using GraphPad Prism version 5 (GraphPad Prism Software, Inc, San Diego, CA, USA) and data were presented using median and range or mean ± SD. Mann–Whitney tests were used for comparisons between the two groups. For comparisons between more than two groups, nonparametric Kruskal–Wallis tests were performed followed by Dunn’s multiple comparison tests as a post hoc test. The comparisons were considered statistically significant when the *P* value was less than 0.05 (*P* < 0.05).

## Results

### Optimization of PCR efficiency

**Fig. 1:**
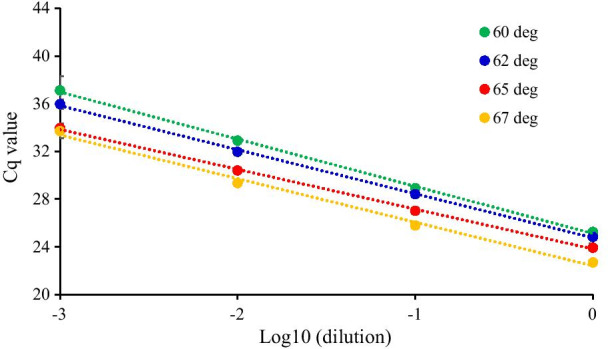
Standard curves of synthetic miRNA-16 according to annealing temperature. The Cq values were plotted against log of four different ten-fold cDNA dilutions (1, 10, 100, and 1000) generated from synthetic miRNA-16. This series of four dilutions was measured in three replicates per dilution

**Fig. 2:**
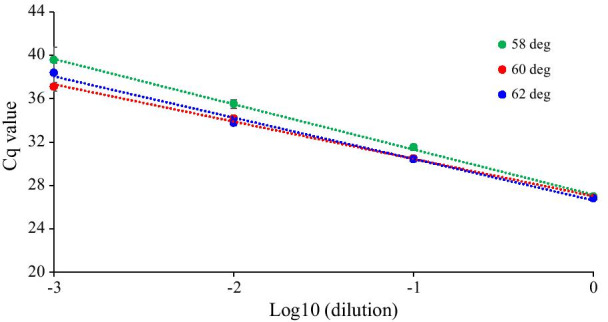
Standard curves of synthetic Cel-miRNA-39-1 according to annealing temperature. The Cq values were plotted against log of four different ten-fold cDNA dilutions (1, 10, 100, and 1000) of generated from synthetic Cel-miRNA-39-1. This series of four dilutions was measured in three replicates per dilution

**Fig. 3:**
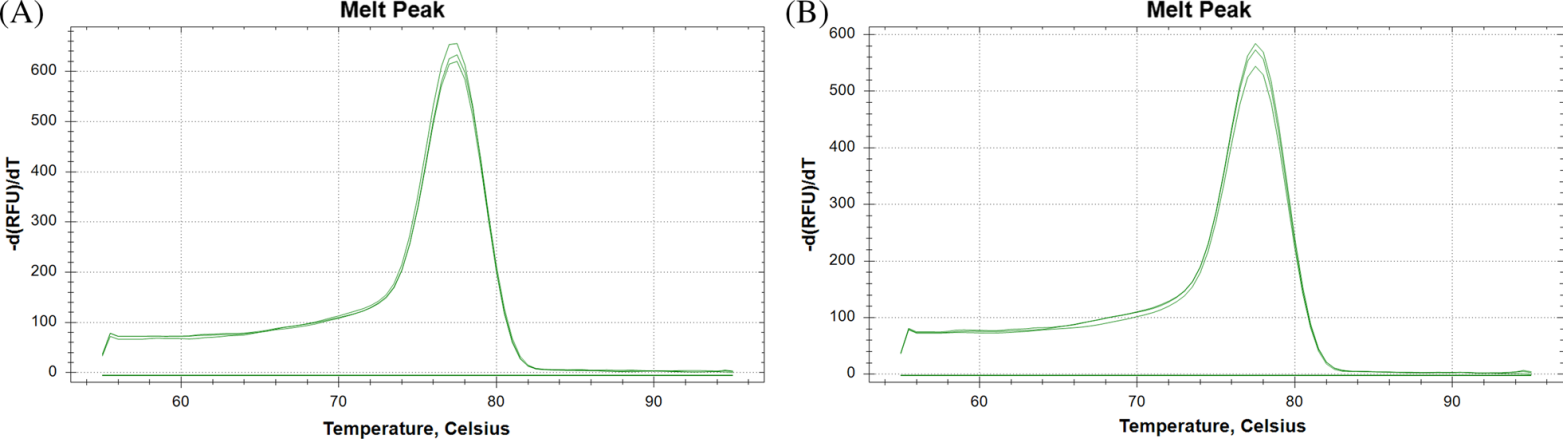
Melt curve of synthetic Cel-miR-39-1 (**A**) and synthetic miRNA-16 (**B**). Melting curve analysis was performed from 60 to 95 °C for Cel-miRNA-39-1 and from 65 to 95 °C for miRNA-16

PCR efficiency was first evaluated with respect to the annealing temperature using miRNA-16 and cel-miRNA-39 as endogenous and exogenous miRNA, respectively. The amplification efficiency for the reaction conditions should first be determined when setting up a PCR assay. Acceptable amplification efficiencies usually range from 90 to 110% and indicate that PCR conditions, such as primer design, reaction conditions, and annealing temperatures, are appropriate for yielding accurate qRT-PCR results [8]. For the optimization of the annealing temperature for miRNA-16 and cel-miRNA-39, standard curves were constructed by plotting Cq values against the logarithmic dilution factors using five and four tenfold serial dilutions of cDNA templet generated from synthetic miRNA-16 and Cel-miRNA-39-1, respectively as shown in Figs. 1 and 2. Amplification efficiencies and R^2^-values was estimated through the linear regression of standard curves. For miRNA-16, as shown in Fig. 1 and Table 1, the Cq value decreased as annealing temperature increased from 60 °C to 67 °C and R^2^-values were higher than 0.99, indicating high linearity between PCR-signal and standards concentration at all tested annealing temperatures. The mean slope and amplification efficiency was from − 3.959 to − 3.346 and from 78.9 to 99.0%, respectively. A temperature of 65 °C resulted in the lowest Cq value and the best amplification efficiency with the 95% confidence interval (CI) being 80.8–117.9. Like standard curve about miRNA-16, for cel-miRNA-39, R^2^-values were also higher than 0.99. The change of the Cq value was not large; however, mean slope was from − 3.959 to − 3.346 and mean amplification efficiencies varied from 73.6 to 95.1% for the tested annealing temperatures (58–62 °C). At 60 °C, the amplification efficiency was highest with the 95% CI being 81.1–105.5. (Fig. 2, Table 2). Moreover, single peaks were observed in melting curves at these annealing temperatures, indicating a high specific amplification (Fig. 3). Based on these results, the optimized annealing temperatures were 65 °C and 60 °C for miRNA-16 and Cel-miRNA-39–1, respectively. Using these optimized annealing temperatures, the effects of contaminants on the amplification efficiency were further evaluated to optimize the extraction of miRNAs from plasma.

**Table 1:** Comparison of slop, amplification efficiency, and R^2^ estimated through the linear regression of triple standard curves of miRNA-16 according to the annealing temperature

Parameters	Annealing temp. (℃)				
	60	62		65	67
Slope					
Median	− 3.363	− 3.751	− 3.380		− 3.724
Range	− 4.3555 to − 3.6579	− 3.8144 to − 3.5217	− 3.5037 to − 3.1532		− 3.7350 to − 3.5115
95% CI	− 4.849 to − 3.068	− 4.078 to − 3.318		− 3.787 to − 2.904	− 3.970 to − 3.344
Efficiency (%)					
Median	81.48	84.76		97.62	85.57
Range	69.7 to 81.5	82.9 to 92.3		92.9 to 107.6	85.2 to 92.7
95% CI	56.89 to 102.3	74.27 to 99.01		80.82 to 117.9	77.42 to 98.23
R^2^					
Median	0.9985	0.9995		0.9987	0.9914
Range	0.9902 to 0.9996	0.9966 to 0.9995		0.9981 to 0.9998	0.9914 to 0.9995
95% CI	0.9833 to 1.009	0.9943 to 1.003		0.9967 to 1.001	0.9966 to 1.009

**Table 2:** Comparison of slop, amplification efficiency, and R^2^ estimated through the linear regression of triple standard curves of Cel-miRNA-39-1 according to the annealing temperature

Parameters	Annealing temp. (℃)		
	58	60	62
Slope			
Median	− 4.144	− 3.554	− 3.648
Range	− 4.5042 to − 3.8800	− 3.5939 to − 3.3490	− 4.1735 to − 3.6066
95% CI	− 4.953 to − 3.396	− 3.825 to − 3.173	− 4.594 to − 3.024
Efficiency (%)			
Median	74.40	91.15	62.01
Range	66.7 to 81.0	89.8 to 98.9	73.6 to 89.3
95% CI	56.29 to 91.81	81.08 to 105.5	62.01 to 105.3
R^2^			
Median	0.9970	0.9993	0.9962
Range	0.9820 to 0.9993	0.9907 to 0.9999	0.9899 to 0.9973
95% CI	0.9696 to 1.016	0.9838 to 1.009	0.9845 to 1.004

### MiRNA extraction from plasma

Owing to its high sensitivity, qRT-PCR is the most widely used method for miRNA analyses. However, the accuracy of qRT-PCR assays is affected by interference caused by various contaminants, including extraction reagents and sample matrix [6]. Therefore, miRNA pre-analytical methods are usually focused on eliminating interfering substances from extracted miRNAs. However, for the analysis of circulating miRNAs in plasma by qRT-PCR, it is necessary to improve the miRNA yield and minimize the loss of extracted miRNAs with the removal of PCR inhibitors because the concentrations of miRNAs in plasma are substantially lower than those in cells or tissues [9–11]. To develop a method for reliable and reproducible PCR analyses, the pre-analytical method for miRNA extraction from plasma was optimized with respect to amplification efficiencies and Cq values.

Various steps during extraction with TRIzol were evaluated for purity and recovery, including the collection of the aqueous phase, chloroform extraction, and ethanol washing. The optimization of these steps is a major issue for the extraction of high-quality miRNAs in high quantities from various samples, including plasma. When the upper aqueous phase is collected after phase separation, unwanted contaminants and sample matrix in the lower phase or interphase may be transferred. These contaminants and sample matrix may interfere with subsequent PCR. Accordingly, this step is critical to ensure the quality of isolated RNA. It is common to leave 50–100 μL of the aqueous phase. However, this may cause a substantial loss of RNA. Therefore, the appropriate transfer volume was optimized with respect to the extraction quantity and quality.

**Fig. 4:**
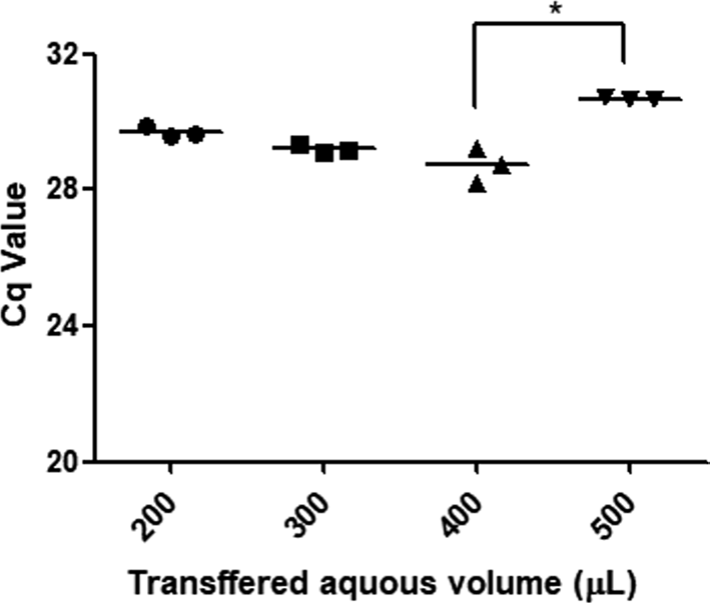
Efficiency of RNA extraction for endogenous miRNA-16 detection from plasma by qRT-PCR in different transferred upper phase volumes. Results represent the raw Cq values obtained for miRNA-16. RNA samples from plasma were obtained by extraction with Trizol LS in single chloroform extraction and without ethanol washing. In the figure, dots represent individual Cq values, the horizontal bar is the group mean. Statistical differences were determined using Kruskal–Wallis test followed by Dunn’s post hoc test for multiple comparisons (*P = 0.0216)

In this study, different volumes of the upper layer (200, 300, 400, and 500 μL) were transferred to new tubes after phase separation and the addition of chloroform to the TRIzol LS solution. The removal of contaminants and extraction yield were evaluated by measuring the amplification efficiency and Cq values of endogenous miRNA-16. For the evaluation of amplification efficiency of extracted endogenous miRNA-16 from plasma, standard curves were constructed by the linear regression line of Cq values against the logarithmic dilution factors using five tenfold serial dilutions of cDNA templet generated from extracted RNA samples. The intensity of amplified miRNA-16 initially increased (lower Cq value) as the volume of the upper layer increased; however, the intensity began to decrease when using 500 μL of the upper layer (higher Cq value) (Fig. 4) and showed significant lower than when 400 μL of the aqueous phase was transferred. In addition, when 200 to 400 μL of the upper layer was transferred, the amplification efficiency was 102.8–105.6%, which is within the recommended range (90–110%). However, when 500 μL of the aqueous phase was transferred, the amplification efficiency was 114.5 ± 11.6%, indicating interference. These results indicate that contaminant interference did not substantially influence the amplification efficiency for volumes ≤ 400 µL, and the extracted miRNA quantity also increased with increasing volumes of aqueous phase. However, when 500 µL was transferred, moderately high interference caused by sample matrix contaminants taken from the interphase was detected. As a result, 400 µL, which is about 50% of the TRIzol volume used in the extraction step, was identified as the optimal volume of the upper layer for transfer after phase separation. This volume maximizes the quality and quantity of extracted RNA.

Most RNA extraction kits, including TRIzol LS, are based on phenol, which can interfere with miRNA analyses by qRT-PCR. An ethanol washing step is therefore normally included in the TRIzol extraction process. This step also removes residue from the extraction reagents, such as phenol and salts, which are known to interfere with qRT-PCR. An additional chloroform extraction step is often used with ethanol washing to remove residual phenol and increase the purity of RNA [12].

In this study, the effects of single and triple chloroform extraction with or without ethanol washing on the quality and quantity of extracted RNA with the removal of residual phenol were evaluated. The effect of phenol removal was first observed at 260 nm using the NanoDrop One built with the Acclaro Contaminant Identification (ID) feature. Residual phenol after miRNA extraction can be detected by absorbance at 260 nm because phenol shows strong absorption signals near 270 nm. As shown in Table 3, residual phenol and the concentration of extracted RNA decreased with ethanol washing and the addition of chloroform extraction. The reduction in the amount of extracted RNA with ethanol washing was greater than that caused by the addition of a chloroform extraction step. The effects of these additional chloroform extraction and ethanol washing steps were also observed in the qRT-PCR analysis. The removal of interference and recovery of miRNAs in each case was estimated with respect to Cq values and amplification efficiency.

**Table 3:** Effect of multiple chloroform extraction and ethanol washing on elimination of RNA yield and phenol (n = 3)

Extraction and washing condition	RNA concentration(ng/µL) Median (range)	Detected phenol contaminant at 260 nm Median (range)
Once chloroform extraction without EtOH washing	294.0 (240.7–381.45)	6.44 (5.15–8.23)
Once chloroform extraction and EtOH washing	152.4 (150.0–175.9)	3.43 (2.13–4.00)
Ttriple chloroform extraction without EtOH washing	201.3 (158.0–215.0)	4.00 (2.94–4.08)
Triple chloroform extraction and EtOH washing	130.2 (102.8–158.1)	2.63 (2.07–2.95)

**Fig. 5:**
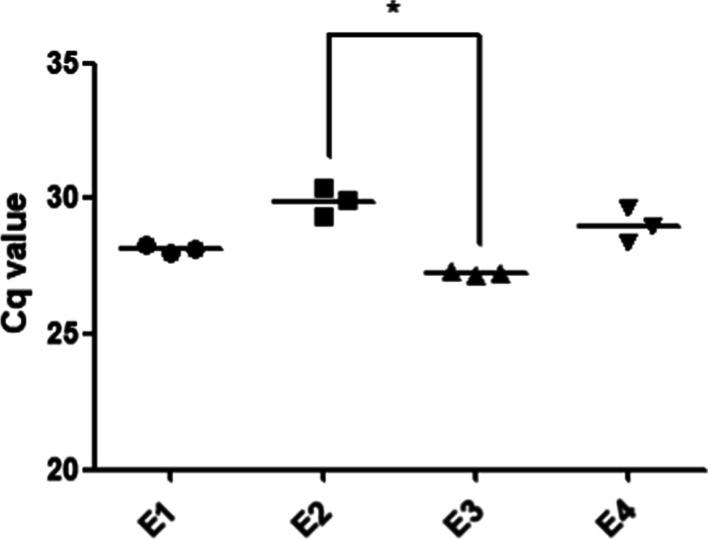
Efficiency of RNA extraction for endogenous miRNA-16 detection from plasma by qRT-PCR with number of chloroform extractions and with or without ethanol washing. Results represent raw Cq values obtained for miRNA-16. RNA samples from plasma were obtained by extraction with Trizol LS in transferring 400 uL of upper phase after phase separation. E1: Once chloroform extraction without EtOH washing, E2: Once chloroform extraction with EtOH washing, E3: Triple chloroform extraction without EtOH washing, E4: Triple chloroform extraction with EtOH washing. In the figure, dots represent individual Cq values, the horizontal bar is the group mean. Statistical differences were determined using Kruskal–Wallis test followed by Dunn’s post hoc test for multiple comparisons (*P = 0.0289)

As shown Fig. 5, Cq values for miRNA-16 were lower for triple chloroform extraction than for single chloroform extraction. This indicates that the detection sensitivity of miRNA-16 increased by the removal of additional interference by multiple chloroform extractions. Therefore, two additional chloroform extractions were included in the conventional extraction process using TRIzol LS to reduce interference and increase miRNA detection sensitivity. With ethanol washing, Cq values for miRNA-16 were slightly higher than those obtained without ethanol washing. This may be explained by a trade-off between the elimination of interfering substances and the loss of extracted RNA. Moreover, the combination of triple chloroform extraction and ethanol washing resulted in the extensive loss of extracted RNA. The triple chloroform extraction without ethanol washing had the lowest Cq value and significantly improved intensity compared to the typical miRNA extraction process using single chloroform extraction and ethanol washing. These results suggest that triple chloroform extraction effectively minimizes contaminants, resulting in improved sensitivity of miRNA analysis by qRT-PCR. Ethanol washing resulted in the extensive loss of extracted RNA in addition to a reduction in contaminants. Therefore, in subsequent analyses, miRNAs were extracted from plasma using TRIzol LS with a triple chloroform extraction process without ethanol washing. The PCR efficiency was 91.1–106.0%, an acceptable range, and was not influenced by multiple chloroform extractions and ethanol washing. This is likely because contaminants affecting the amplification efficiency were removed by optimizing the transfer upper volume during phase separation.

### Application

Using the optimized contaminant elimination and qRT-PCR conditions, RNA was extracted from plasma or serum samples and screened for candidate miRNA biomarkers for AMI. Many studies have characterized circulating miRNAs in the plasma or serum of patients with AMI, and there is increasing evidence for the diagnostic and prognostic value of miRNAs in AMI [13, 14]. However, further studies of candidate miRNAs are needed before their practical application as biomarkers for the diagnosis of AMI. In this study, five miRNA candidates were screened in human plasma samples from four patients with AMI and three control adults. The five miRNAs are related to AMI based on a comprehensive review of the literature. Four miRNAs (miRNA-16 [15, 16], -21 [17, 18], -132 [19–21], and -155 [22, 23]) have been identified as biomarker candidates for AMI but remain controversial owing to inconsistent results among studies. miRNA-499 expression levels are higher in tested all AMI plasma samples than in samples from control individuals; this miRNA is an established diagnostic biomarker for AMI [24, 25].

**Fig. 6:**
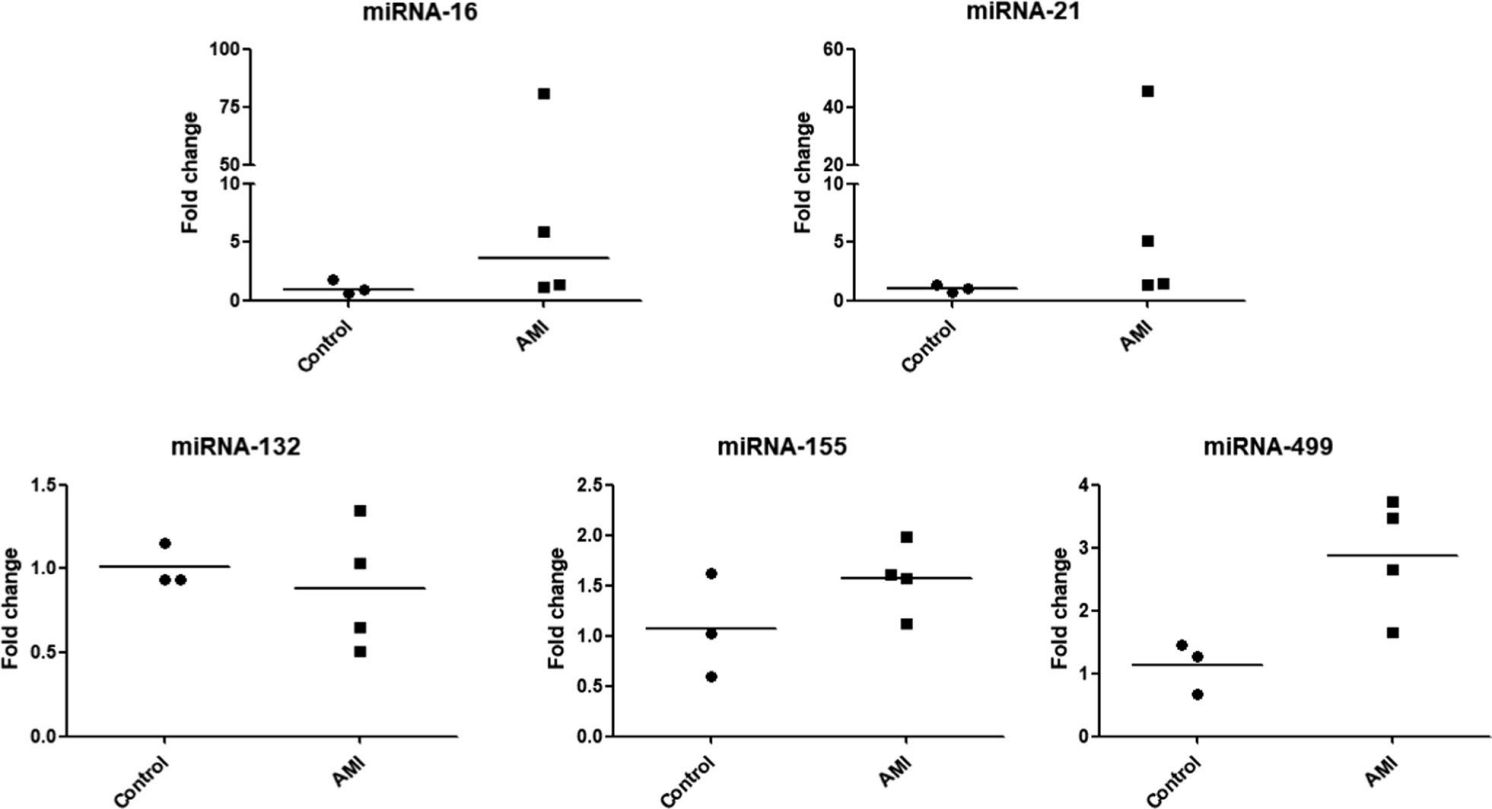
Relative levels of miRNA-16, miRNA-21, miRNA-132, miRNA-155, and miRNA-499 analyzed by qRT-PCR in four AMI patients vs three controls. Relative level of each miRNA was obtained by normalization of Cel-miRNA-39-1, and horizontal middle line in each data set represents mean. Mann–Whitney test was performed comparing AMI patients with control miRNAs levels. The *P* values was 0.2286 for miRNA-16, *P* = 0.1143 for miRNA-21, 0.8571 for miRNA-132, 0.4000 for miRNA-155 and 0.0571 for miRNA-499

First, the amplification efficiency of the selected miRNAs was evaluated. The amplification efficiency with the selected annealing temperature was high, as shown in Table 4. Then, samples were screened using our optimized extraction and PCR conditions. As shown in Fig. 6, the expression level of miRNA-499 was about 2.88-fold higher in patient samples than in control samples, as expected. Among other tested miRNAs, miRNA-132 and miRNA-155 showed differences in expression between the control and AMI groups. For miRNA-16 and miRNA-21, high individual differences were observed in AMI groups. The miRNA levels were 80.9- and 45.6-fold higher in some patient samples than in control samples, respectively. In the AMI group, mean miRNA-132 levels were lower than those in the control group, consistent with previous results [19]; however, the difference was small and observed individual difference. The mean expression level of miRNA-155 in the AMI group was 1.54-fold higher than that in the control group, similar to previous results [22, 23], although the difference was not significant.

**Table 4:** Amplification efficiency of tested miRNAs at optimized annealing temperature (n = 3)

miRNAs	Annealing temperature (°C)	Median Efficiency (%)	Range	95% CI
Cel-miRNA-39-1	60	91.2	99.0–89.8	81.1–105.4
miRNA-16	65	97.6	107.6–93.0	80.9–117.9
miRNA-21	65	98.1	106.3–96.4	87.1–113.4
miRNA-132	60	95. 2	95.8–85.2	77.3–106.9
miRNA-155	65	108.7	112.7–86.5	67.6–137.7
miRNA-499	65	94.9	94.6–95.4	93.9–96.0

## Discussion

In a PCR analysis, the number of target sequence molecules should double during each replication cycle, corresponding to 100% amplification efficiency. However, in practice, inappropriate reaction conditions and polymerase inhibition can affect primer template annealing and can result in poor amplification, affecting the interpretation of results [6, 7]. Assessments of amplification efficiency can provide information on inappropriate reaction conditions and the presence of contaminants interfering with qRT-PCR [26]. Reliable qRT-PCR assays of miRNAs require optimized PCR and extraction conditions based on the amplification efficiency. In general, inappropriate reaction conditions can be caused by suboptimal annealing temperatures or pipetting errors, and polymerase inhibition by contaminants transferred from the RNA isolation process or sample matrix [6, 7]. Therefore, we used pooled plasma for PCR and extraction condition evaluation to perform miRNA analysis in plasma while minimizing the individual differences in plasma components although the difference of miRNA profile may observe due to individual variation between plasma components in screening of miRNA from real patient and normal plasma samples.

In this study, we determined extraction conditions to minimize interference caused by contaminants for qRT-PCR assays of endogenous miRNAs in pooled plasma samples because endogenous plasma miRNA levels are substantially lower than levels in cells or tissues, RNA extraction conditions were also optimized with respect to RNA yields. The tested extraction conditions including the volume of the upper layer transferred after phase separation, multiple chloroform extractions, and ethanol washing. The amplification efficiency and Cq values were assessed to evaluate the effects on endogenous miRNA analyses in plasma.

The amplification efficiency varied according to the annealing temperature, resulting in variation in Cq values (Figs. 1, 2, Tables 1, 2). Among the extraction processes considered in the analysis, the volume of the upper layer transferred after phase separation had the greatest effect on amplification efficiency because sample matrix and residual reagent were included in the upper phase. Multiple chloroform extractions and ethanol washing had greater effects on Cq values than on the amplification efficiency. This is likely because the appropriate volume of the upper layer was obtained after phase separation to minimize interference affecting the amplification efficiency. Multiple chloroform extractions or ethanol washing appear to lower Cq values by removing additional residual reagents, such as phenol. Multiple chloroform extractions had a greater effect on contaminants than on RNA loss, thus lowering the Cq value, whereas ethanol washing reduced both interference and RNA yields. Therefore, higher Cq values were observed with ethanol washing than with chloroform extraction, as shown Fig. 5. These effects of multiple chloroform extractions or ethanol washings on the removal of RNA and phenol were also observed by spectrophotometry (Table 3). By applying only triple chloroform extraction without ethanol washing, interfering contaminants were reduced, while maximizing RNA yields, resulting in a low Cq value. Indeed, in the analysis of endogenous miRNAs in plasma, prior to optimization, the Cq values for the tested miRNAs were similar (i.e., around 30–32) and the Cq values for miRNA-499 were around 35–37, which were acceptable (data not shown). However, under the optimized extraction and qRT-PCR conditions, the Cq values for the tested miRNAs in plasma were 26–34. These values were sufficiently low and variable to evaluate endogenous miRNAs in plasma samples.

Finally, for reliable qRT-PCR assays of miRNAs, it was necessary to optimize the annealing temperature and to transfer an appropriate volume of the upper layer after phase separation. This was followed by triple chloroform extraction without ethanol washing. In addition, our optimized extraction conditions were successfully applied to screen miRNAs in plasma as biomarker candidates for the diagnosis of AMI.

This was a pilot study aimed at determining the feasibility of reliable analyses of endogenous miRNAs in plasma using optimized sample extraction and qRT-PCR conditions.

For this purpose, optimized sample extraction and qRT-PCR conditions were applied to determine whether levels of previously reported miRNA biomarker candidates, including miRNA-499, differ between plasma samples from patients and control individuals. We also included miRNA-499, an established diagnostic biomarker for AMI [24, 25], to determine the reliability of miRNA screening using the optimized method. Levels of miRNA-499 were higher in the plasma of patients with AMI than in control individuals, as expected. Further, mean miRNA-155 levels were 1.5-fold higher in the AMI group than in the control group and the individual difference was small.

Although this study is a pilot study but has two major limitations. In this study, to identify miRNA related AMI, samples from patients with diseases other than AMI were excluded from this experiment because one miRNA is associated with a variety of diseases. Many AMI patients recruited had diseases, such as hypertension, diabetics and dyslipidemia and it was impossible to select enough many samples to even out bias. In addition, because heart disease is age-related, the study was restricted to men aged 40–60 years to eliminate the impact of these confounding variables. For these reasons, the sample size was very small, which limits the evaluation and identification of miRNAs biomarker candidates for AMI. A sample size that is too small affects the efficiency of the study and leads to the loss of the ability to validate a possibly significant effect, and the study will have insufficient power to detect the true difference between the groups statistically.

Another limitation of our study was small sample volume, which didn’t perform replicate analyzes in other laboratories to evaluate the reliability of our biomarker screening results. Analysis of miRNAs shows inconsistent results between studies due to PCR inhibitor and low level of miRNA in plasma sample. Replication of analysis and measurements through different personnel or laboratory is important to reporting reliability of the analysis miRNA from plasma using PCR.

In our study, we observed miRNA candidate that differed between AMI patients and normal subjects, but sample size was too small to obtain sufficient results to assess statistical significant. Also, in order to obtain our reliable biomarker screening results, extraction and detection conditions were evaluated and optimized through PCR efficiency evaluation, but the reproducibility of the results was not confirmed because repeated analysis by other laboratories or personnel was not performed due to the limited sample volume. Therefore, we suggest that tested miRNA candidates should be further explored as diagnostic tools in AMI through performing validation and reproducibility of measurement with sufficient sample size and volume by broadening the age range of the patients and normal group and excluding sex from the confounders in order to draw reliable conclusions in the future.

## Conclusion

This was a pilot study aimed at optimizing sample extraction and qRT-PCR conditions for reliable endogenous miRNA analyses in plasma. In this study, we have optimized extraction and qRT-PCR conditions, while improving miRNA yields and minimizing the loss of extracted miRNA by evaluations of the amplification efficiency. The optimal extraction and PCR conditions were applied to detect miRNAs in plasma samples from control individuals and patients with AMI in order to evaluate candidate diagnostic biomarkers.

Our results provide a basis for reliable and reproducible miRNA analyses in plasma by qRT-PCR when TRIzol reagent is used for miRNA extraction. To our knowledge, this is the first analysis of the effects of the upper layer volume, multiple chloroform extractions, and ethanol washing on contaminants, extraction efficiency, and miRNA yields determined by amplification efficiency in the qRT-PCR system.

## Data Availability

The datasets used and/or analysed during the current study available from the corresponding author on reasonable request.

## Abbreviations

miRNA: MicroRNA
tRNA: Transfer RNA
qRT-PCR: Quantitative real-time PCR
cDNA: Complementary DNA
Cq: Quantification cycle
AMI: Acute myocardial infarction
CK-MB: Creatine kinase-myocardial band
LBBB: Left bundle branch block
ECG: Electrocardiogram
CVD: Cardiovascular disease
